# Colorectal cancer incidence and mortality trends by sex and population group in South Africa: 2002–2014

**DOI:** 10.1186/s12885-021-07853-1

**Published:** 2021-02-06

**Authors:** Lactatia Motsuku, Wenlong Carl Chen, Mazvita Molleen Muchengeti, Megan Naidoo, Tamlyn Mac Quene, Patricia Kellett, Matshediso Ivy Mohlala, Kathryn M. Chu, Elvira Singh

**Affiliations:** 1 National Cancer Registry, National Health Laboratory Service, 1 Modderfontein road, Sandringham, Johannesburg, 2131 South Africa; 2 Department of Global Health, South African Centre for Epidemiological Modelling and Analysis (SACEMA), Stellenbosch University, Stellenbosch, South Africa; 3 Faculty of Health Sciences, Sydney Brenner Institute for Molecular Bioscience, University of the Witwatersrand, Johannesburg, South Africa; 4 Faculty of Health Sciences, School of Public Health, University of the Witwatersrand, Johannesburg, South Africa; 5 Department of Global Health, Centre for Global Surgery, Stellenbosch University, Cape Town, South Africa

**Keywords:** Colorectal cancer, Incidence, Mortality, South Africa

## Abstract

**Background:**

South Africa (SA) has experienced a rapid transition in the Human Development Index (HDI) over the past decade, which had an effect on the incidence and mortality rates of colorectal cancer (CRC). This study aims to provide CRC incidence and mortality trends by population group and sex in SA from 2002 to 2014.

**Methods:**

Incidence data were extracted from the South African National Cancer Registry and mortality data obtained from Statistics South Africa (STATS SA), for the period 2002 to 2014. Age-standardised incidence rates (ASIR) and age-standardised mortality rates (ASMR) were calculated using the STATS SA mid-year population as the denominator and the Segi world standard population data for standardisation. A Joinpoint regression analysis was computed for the CRC ASIR and ASMR by population group and sex.

**Results:**

A total of 33,232 incident CRC cases and 26,836 CRC deaths were reported during the study period. Of the CRC cases reported, 54% were males and 46% were females, and among deaths reported, 47% were males and 53% were females. Overall, there was a 2.5% annual average percentage change (AAPC) increase in ASIR from 2002 to 2014 (95% CI: 0.6–4.5, *p*-value < 0.001). For ASMR overall, there was 1.3% increase from 2002 to 2014 (95% CI: 0.1–2.6, p-value < 0.001). The ASIR and ASMR among population groups were stable, with the exception of the Black population group. The ASIR increased consistently at 4.3% for black males (95% CI: 1.9–6.7, *p*-value < 0.001) and 3.4% for black females (95% CI: 1.5–5.3, *p*-value < 0.001) from 2002 to 2014, respectively. Similarly, ASMR for black males and females increased by 4.2% (95% CI: 2.0–6.5, *p*-value < 0.001) and 3.4% (, 95%CI: 2.0–4.8, p-value < 0.01) from 2002 to 2014, respectively.

**Conclusions:**

The disparities in the CRC incidence and mortality trends may reflect socioeconomic inequalities across different population groups in SA. The rapid increase in CRC trends among the Black population group is concerning and requires further investigation and increased efforts for cancer prevention, early screening and diagnosis, as well as better access to cancer treatment.

## Background

Colorectal cancer (CRC) is regarded as a leading cause of cancer morbidity and mortality worldwide [1]. It is ranked third in cancer incidence and second in cancer mortality globally [1]. In 2019, there were 1.8 million new CRC cases and 880,792 CRC deaths worldwide [1–3]. In 2018, the age-standardised incidence rate (ASIR) and age-standardised mortality rate (ASMR) in the African region were estimated at 8.2 and 5.6 per 100,000 population, respectively [4]. According to the Globocan report, the estimated ASIR and ASMR for South Africa (SA) in 2018 were 14.4 and 7.6 per 100,000, respectively [5]. The latest South African National Cancer Registry (NCR) 2016 report observed 3884 cases of CRC with an estimated 6.81 and 11.01 ASIR per 100,000 population for females and males, respectively [6]. There is limited data on colorectal cancer survival in SA [7, 8]. The patient survival is dependent on multiple factors such as the topography, morphology, staging and treatment type [7]. According to the CONCORD-3 study for the period from 2010 to 2014 among adults aged 15–99 years, the 5-year survival rate in SA was less than 20% [9].

Disparities in CRC between countries exist based on the Human Development Index (HDI). HDI is a composite score of life expectancy, education and per capita income of countries [10]. Countries with a high HDI reported the highest CRC incidence rates, while low HDI countries reported the highest CRC mortality rates [1]. The CRC incidence of a country increases with increasing HDI status and, as such, may be used to signal changes in socioeconomic status [1]. The rise in incidence rates in countries undergoing rapid developmental transition may be attributed to changes in diet, obesity, and other lifestyle factors such as higher alcohol and red meat consumption [1, 11, 12]. On the other hand, the increase in CRC mortality rates, observed in low HDI countries, may be attributed to minimal or lack of appropriate screening and early detection programmes, and poor access to cancer treatment [11].

The HDI of SA has been increasing steadily since 1990 [13]. Despite an increasing HDI, the Gini coefficient in SA is 0.63, indicating considerable societal inequality [13, 14]. The Gini coefficient measures wealth distribution and ranges from 0 to 1, where 0 represents perfect equality and 1 indicates perfect inequality. The inequity in socioeconomic status and health access, among other factors, influences the health outcomes of the SA population [15–17]. As a result of the unique political history of SA, many of these inequalities are apparent when examining health trends by population groups (Black, White, Asian, and Mixed race-commonly known as Coloured in SA). To appropriately plan for interventions, prevention, and control of CRC in the era of epidemiologic and economic transition, the evaluation of CRC patterns across population groups, and sex are necessary. This study aims to provide insights into the CRC incidence and mortality trends by population group and sex for SA.

## Methods

### Study design and data sources

A cross-sectional study was conducted using secondary data analysis of two datasets, to determine incidence trends and mortality trends for CRC between 2002 and 2014 in SA by sex and population groups.

To determine incident CRC trends, the NCR database was used. The NCR methodology is outlined in detail by Singh et al. [18]. Briefly, the NCR collects data on all pathologically confirmed cancer cases from both public and private laboratories across SA. The NCR then collates, analyses, interprets and reports annual cancer incidences by age groups, population groups, and sex. All cancers are coded according to the International Classification of Diseases for Oncology, 3rd edition (ICD-O-3) [19].

The cancers reported by NCR are primary incident cancers. Cancers reported in metastatic sites (for example, lymph nodes) are investigated to determine the primary tumour site and are registered with the primary tumour site topography. If a primary site cannot be determined, the tumour is registered as an unknown primary site. The CRC primary incident cases, defined as ICD-O-3 codes C18, C19, and C20, from 2002 to 2014, were extracted from the NCR.

The variables extracted included the year of diagnosis, sex, population group, age at diagnosis, and morphology and topography of cancer.

CRC deaths from 2002 to 2014 were extracted from the Statistic South Africa (STATS SA) mortality and causes of death database. STATS SA collates national mortality statistics for the registered causes of deaths from death certificates. We extracted the date of death, sex, population group, smoking status, marital status, education level, and age of the deceased for individuals whose first or underlying causes of death were recorded as CRC (ICD-O-3 codes C18, C19, and C20).

### Statistical analysis

Stata® statistical software version 14.2 (StataCorp LLC, Texas, USA) was used to generate frequencies and calculate ASIR and ASMR by sex and population group. The estimated mid-year population from STATS SA was used as the denominator. The Segi world standard population was used for age standardisation. The Segi world standard population was developed by Dr. Mitsuo Segi in the late 1950s to ensure international comparison of rates and evaluation of changes in incidence by comparing the current rates with the past published rates [20–22]. It was later modified in 1966 by Doll et al. [20]. It is the most commonly used world standard population, particularly in the field of cancer and allows in-country and between-country comparisons [21, 23, 24]. A detailed description of the calculation of the ASIR and ASMR is available elsewhere [20]. Briefly, the crude incidence rates were calculated first by dividing the total number of new primary CRC cases/deaths observed in a particular year by the total number of population (STATS SA mid-year population) in that same year, stratified by age groups, sex and population group. The crude rates were multiplied by the weighted Segi world standard population to obtain age-standardised incidence or mortality rates. The weighted Segi world standard population is calculated by dividing the Segi world standard population for each age group (5-year intervals, e.g. 0–5 years) by the total Segi world standard population of all age groups.

The Calculation methods are as follows:

$$ \mathrm{Crude}=\frac{\mathrm{Number}\ \mathrm{of}\ \mathrm{new}\ \mathrm{cases}/\mathrm{deaths}}{\mathrm{Mid}-\mathrm{year}\ \mathrm{population}}\times 100,000 $$

$$ \mathrm{WSP}\ \mathrm{weighting}=\frac{\mathrm{WSP}\ \left(\mathrm{for}\ \mathrm{each}\ \mathrm{age}\ \mathrm{group}\right)}{\mathrm{Total}\ \mathrm{WSP}\ \mathrm{for}\ \mathrm{all}\ \mathrm{age}\ \mathrm{group}\mathrm{s}}\times 100,000 $$

$$ \mathrm{ASIR}\ \mathrm{or}\ \mathrm{ASMR}=\mathrm{Crude}\ X\ \mathrm{WSP}\ \mathrm{weighting} $$

The age-standardised rates by year, sex, and population group were imported into Joinpoint regression statistical software for trend analysis for the study period. The model used by Joinpoint statistical software to create the trend patterns was described by Kim et al. [22, 25, 26]. Joinpoint is used to identify points (or “joinpoints”) where there is a significant change in a trend, which enables a more nuanced trend analysis than a standard regression method. Furthermore, Joinpoint is used by the US National Cancer Institute to analyse cancer trends rather than a standard regression method [26]. All parameters were set at default [26]. The method identifies joinpoints based on regression models with zero to five join points. The modelled or estimated annual percentage change was based on the trends within each segment. To quantify for trends over a fixed number of years the average annual percentage change (AAPC) was calculated. The AAPC is a geometric weighted average of the estimated average percentage change trend analysis, with the weights the same as the lengths of each segment during a specified fixed interval. The AAPC was considered significant at the *p*-value threshold of less than 0.05 using a two-sided test.

## Results

### Study population characteristics

A total of 33,232 incident CRC cases were reported during the study period, 56% from private healthcare sector laboratories, and 44% from public healthcare sector laboratories. Of CRC incident cases, 54% were males. Throughout the study period, the annual median incidence was 2292 cases per year (interquartile range (IQR): 2132–3081). The mean age at diagnosis was 61.7 years (±14.1 standard deviation (SD) years) and 61.8 years (±14.7 SD years) for males and females respectively. The White population group had the highest percentage of CRC cases (49%) compared to other population groups. (Table 1).

**Table 1:** Characteristics of Colorectal cancer cases and deaths (2002–2014) in SA

	Colorectal cancer cases, (*N* = 33,232) N(%)	Colorectal cancer death, (*N* = 26,836) N(%)
Age		
Mean (SD)	61.70(±14.38)	65.19 (±14.91)
Age group (years)		
< 15	24 (0.1%)	20 (0.1%)
15–30	894 (2.7%)	551 (2.1%)
31–45	3578 (10.8%)	2272 (8.5%)
46–60	9817 (29.5%)	6447 (24.0%)
> 60	17,760 (53.4%)	16,915 (63.0%)
Missing	1159 (3.5%)	631 (2.4%)
Sex		
Female	15,208 (45.8%)	14,109 (52.6%)
Male	17,995 (54.1%)	12,702 (47.3%)
Missing	29(0.1%)	25 (0.1%)
Population group		
Black	8942 (26.9%)	7163 (26.7%)
Mixed race	4613 (13.9%)	2645 (9.9%)
Asian	1878 (5.7%)	1169 (4.4%)
White	16,207 (48.8%)	10,919 (40.7%)
Missing	1592 (4.8%)	4940 (18.4%)
Laboratory type		
Private	18,666 (56.2%)	–
Public	14,566 (43.8%)	–
Marital status		
Divorced	–	4412 (16.4%)
Married	–	10,630 (39.6%)
Never_Married	–	4887 (18.2%)
Widowed	–	2648 (9.9%)
Missing	–	4259 (15.9%)

There were 26,836 CRC deaths with an annual median of 2138 (IQR: 1982–2321) between 2002 and 2014. Females accounted for 53% of all CRC deaths. The mean age at death was 64 years (±14.9 SD years) and 66 years (±15.7 SD years) for males and females respectively. Sixty-three percent of the deaths were among adults over 60 years of age. The White population group reported the highest proportion of deaths for CRC at 41%, while the Asian population group reported the lowest deaths at 4%. The mortality incidence ratio by age group was highest among the Black population group and lowest among the White population group (data not shown).

### Age-specific incidence and mortality rates

**Fig. 1:**
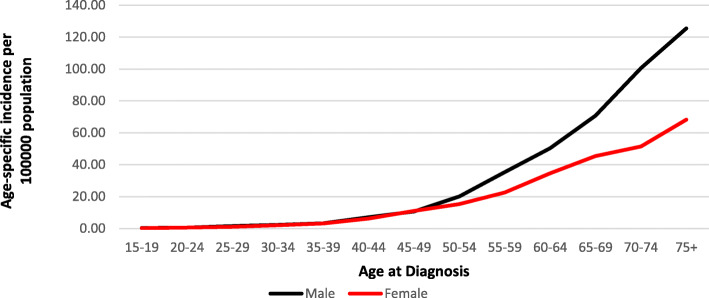
Average age-specific incidence rates, 2002–2014 in SA

**Fig. 2:**
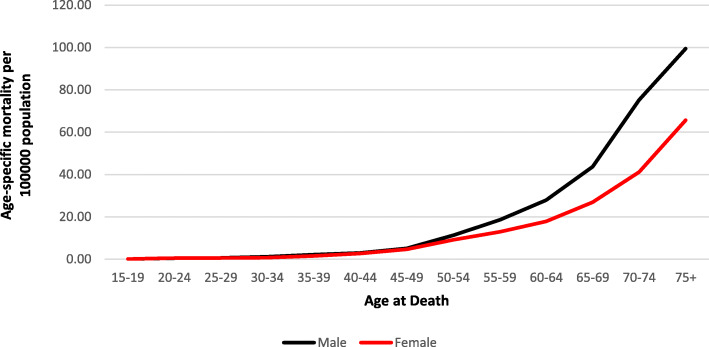
Average age-specific mortality rates, 2002–2014 in SA

Figures 1 and 2 illustrate age-specific incidence and mortality rates for males and females in SA between 2002 and 2014. Rates in males and females increased proportionally until the age of 50 years, after which the rate for males was higher than the rate for females for both incidence and mortality. Rates peaked in the age group of 75 years and older.

### Age-standardised incidence rates trends

**Fig. 3:**
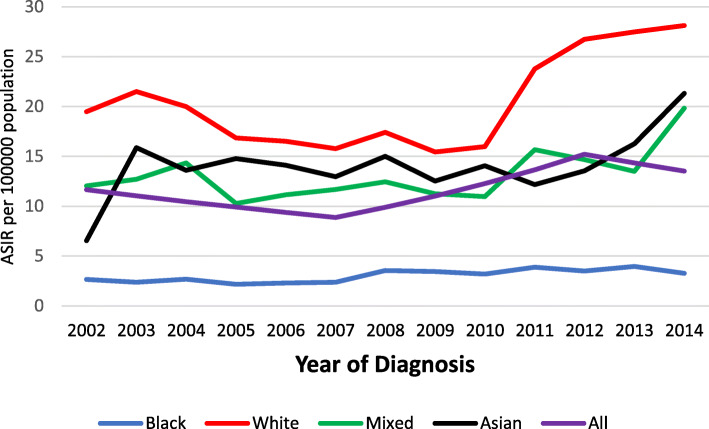
CRC age-standardised incidence rates among males, 2002–2014 in SA

**Fig. 4:**
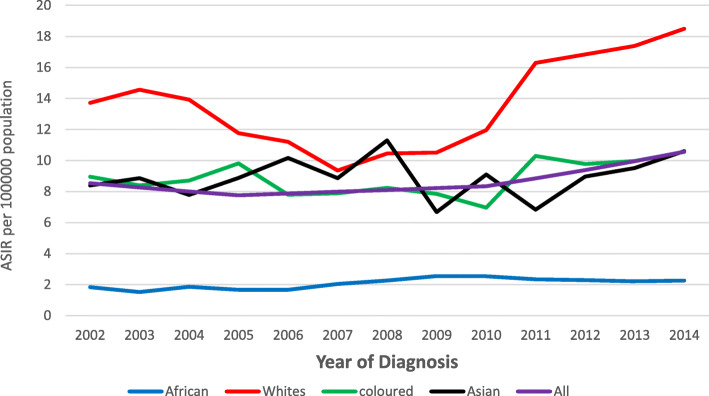
CRC age-standardised incidence rates among females, 2002–2014 in SA

On average, for males and females combined, there was 2.5% annual average increase in ASIR from 2002 to 2014 (annual average percentage change (AAPC) = 2.5, 95% CI: 0.6–4.5, *p*-value < 0.001) (Table 2). ASIR ranged from 11.6 to 13.5 and 8.5 to 10.6 / 100,000 population per year for the study period among males and females, respectively. Overall the ASIR were higher among males compared with females (Figs. 3 and 4).

**Table 2:** Annual Average Percentage Change (AAPC) in CRC incidence and mortality rates by sex and population groups, 2002–2014 in SA

Population group	Sex	Age-standardised Incidence		Age-standardised Mortality	
		Period	AAPC (95%CI)	Period	AAPC (95%CI)
Asian	Males	2002–2014	0.6 (−2.0,3.2)	2002–2014	2.4(−0.4, 5.4)
	Females	2002–2014	3.7 (− 0.2, 7.7)	2002–2014	−0.3(− 2.8, 2.3)
Mixed race	Males	2002–2014	1.2 (−0.8,3.3)	2002–2014	3.0*(0.4,5.7)
	Females	2002–2014	2.5 (−0.0,5.2)	2002–2014	1.3(−0.5, 3.1)
White	Males	2002–2007	−8.8*(− 14.0,-3.2)	2002–2014	0.5(−0.6,1.6)
	Males	2007–2014	10.4*(6.6,14.4)	–	–
	Females	2002–2009	− 3.7 (−8.0, 0.9)	2002–2014	−0.2(−1.3,1.0)
	Females	2009–2014	14.9*(6.4,24.2)	–	–
Black	Males	2002–2014	3.4*(1.5,5.3)	2002–2014	4.2*(2.0,6.5)
	Female	2002–2014	4.3*(1.9,6.7)	2002–2014	3.4*(2.0,4.8)
All	All	2002–2014	2.5*(0.6,4.5)	2002–2014	1.3*(0.1,2.6)

*The average annual percentage change (AAPC) is statistically significant: *p*-value < 0.05

Among males, the highest ASIR was observed among the White population group at 28.12 per 100,000 population in 2014. This rate was 1.3, 1.4, and 8.6 times higher than Mixed race (19.83/100,000), Asian (21.31/100,000), and Black (3.26/100,000) population groups, respectively (Fig. 4). While the CRC ASIR remained stable among males of the Mixed race and Asian population groups, the ASIR among males of the Black population group increased consistently at 3.4% from 2002 to 2014 (AAPC = 3.4, 95% CI: 1.5–5.3, *p*-value < 0.001) (Table 2). Among males of the White population group, a significant decrease of 8.8% was observed from 2002 to 2007 (AAPC = − 8.8, 95% CI: − 14.0- -3.2, *p*-value < 0.001) followed by a 10.4% increase from 2007 to 2014 (AAPC = 10.4, 95%CI: 6.6–14.4, *p*-value < 0.001) (Table 2).

**Fig. 5:**
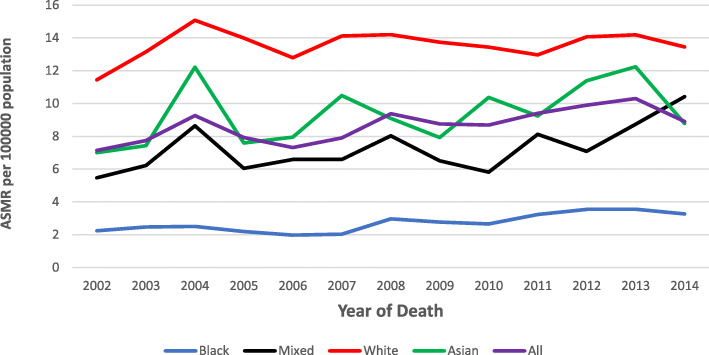
CRC age-standardised mortality rates among males, 2002–2014 in SA

As shown in Fig. 5, the highest ASIR among females were observed in the year 2014 at 18.5 per 100,000 population for the White population group. The ASIR in 2014 were similar for Asian and Mixed race population groups at (10.6/100,000) and (10.6/100,000) respectively. In the same year, Black females reported the lowest ASIR at 2.3 per 100,000 population. The ASIR among females of the Mixed race and Asian population group remained stable, while the ASIR among females of the Black population group increased consistently at 4.3% from 2002 to 2014 (AAPC = 4.3, 95% CI: 1.9–6.7, *p*-value < 0.001). Among females of the White population group, the ASIR trend remained stable until 2009, when ASIR increased by 14.9% from 2009 to 2014 (AAPC = 14.9, 95% CI: 6.4–24.2, *p*-value < 0.01) (Table 2).

### Age-standardised mortality rates trends

**Fig. 6:**
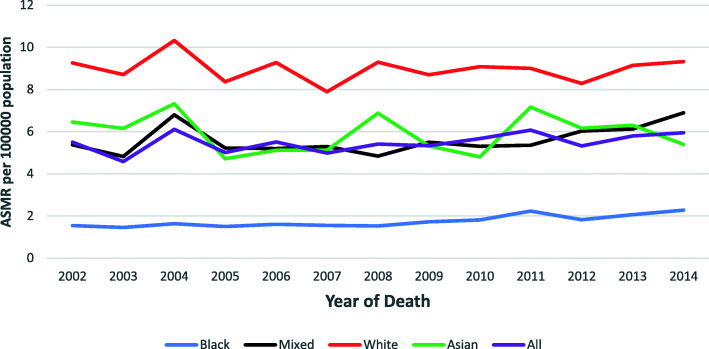
CRC age-standardised mortality rate among females, 2002–2014 in SA

On average, for males and females combined, the ASMR increased by 1.3% from 2002 to 2014 (AAPC = 1.3, 95% CI: 0.1–2.6, *p*-value < 0.01) (Table 2). The overall ASMR ranged from 7.1 to 8.9 and 5.5 to 5.9 /100,000 population per year for the study period among males and females, respectively. Similar to ASIR, the overall ASMR was higher in males than females (Figs. 5 and 6).

The highest ASMR in males was observed in the White population group in 2004 at 15.1 per 100,000 population, and this rate was 1.2, 1.7 and 6.0 times higher than the Asian (12.2/100,000), Mixed race (8.6/100,000), and Black (2.5/100,000) population groups in 2004, respectively (Fig. 5). For males, over the study period, the ASMR remained stable among the White and Asian population groups and increased consistently among the Mixed race and Black population groups by 3.0% (AAPC = 3.0, 95% CI: 0.4–5.7, *p*-value < 0.01) and 4.2% (AAPC = 4.2, 95% CI: 2.0–6.5, *p*-value < 0.01) from 2002 to 2014, respectively (Table 2).

Among females the highest ASMR was reported among the White population group at 10.3 per 100,000 population in 2004, this rate was 1.4, 1.5 and 6.3 times higher than the Asian (7.3/100,000), Mixed race (6.8/100,000) and the Black (1.6/100,000) population groups, respectively (Fig. 6). Among females, a significant change in ASMR was only observed among the Black population group, where the ASMR increased by 3.4% from 2002 to 2014 (AAPC = 3.4, 95% CI: 2.0–4.8, *p*-value < 0.01) (Table 2). The ASMR of females among other population groups remained stable (Table 2).

## Discussion

This is the first study in SA to assess national CRC incidence and mortality trends by population group and sex. We reported overall ASIR from 11.6 to 13.5 and 8.5 to 10.6 per 100,000 population per year for the study period among males and females, respectively. The overall ASMR ranged from 7.1 to 8.9 and 5.5 to 5.9 per100,000 population per year for the study period among males and females respectively. The ASIR and ASMR were highest amongst the White population group and lowest amongst the Black population group. However, the Black population group demonstrated a consistent increase in both ASIR and ASMR for the study period. The age at diagnosis was much closer to the age at death with the average age at diagnosis being 61 years and the average age at death being 65 years. These findings suggest that most cases are diagnosed at a late stage and hence mortality occurs soon after diagnosis and further studies are warranted to delineate this relationship. Screening and early detection of colorectal cancer are recommended as the survival rate is highly dependent on the stage at diagnosis. The age at diagnosis is comparable with other sub-Saharan African countries and the United State of America where most of the cases are diagnosed between the ages of 65 and 68 years [27].

The incidence and mortality were higher in males compared to females. Our study found that there was no significant difference in the pattern of CRC age-specific incidence and mortality rates between males and females. The association between sex and CRC is not fully understood. The disparities found in other studies are partly attributed to differences in exposures and risk factors across sex, such as smoking behaviour and hormones [28–30]. Further studies are needed to understand the relationship between CRC and sex to implement possible interventions to reduce CRC incidence in South Africa.

We found that between 2002 and 2014, the overall ASIR and ASMR increased by 2.5 and 1.3% respectively. As expected for countries undergoing rapid developmental transition and are considered to be medium-to-high HDI, the overall incidence and mortality rates of CRC in SA are increasing with increasing HDI [10, 31–33]. The increase in ASIR is linked to changes in individual behaviour, in particular, lifestyle changes influenced by westernisation [34]. These lifestyle factors include excessive tobacco smoking, excessive alcohol consumption, lack of physical activity leading to obesity, and poor diet i.e. consumption of fast food and red meat, among other factors [35–39]. The increase in ASMR might be associated with quality of care within the country’s health system, for example, inefficient resource allocation (human resources, equipment and medical supplies), lack of access to cancer treatment and or management, and poor health infrastructure may affect mortality rates [7, 35, 40–45]. Other factors include low or lack of awareness, and screening and early detection to detect and remove precancerous polyps as early as possible before they progress into cancerous lesions, as well as late diagnosis, translating into poor prognosis and ultimately increased mortality rates [46–49].

The ASIR and ASMR trends differed across population groups. In 2014, the White population group had the highest ASIR and ASMR, followed by the Asian population group, Mixed race population group, and then the Black population group. The most significant and highest trend changes over time were observed among the Black population group. This is of particular concern as the Black population group comprises 87% of the total SA population [50]. Traditionally, large disparities in access to healthcare exist among the different population groups in SA. For example, the majority of the White population in SA have access to adequate healthcare services in the private healthcare sector, which has more resources [50, 51]. According to the STATS SA general household survey in 2017, 75% of the White population group have private health insurance compared to 10% of the Black population group [52]. Increasing cancer trends and poor access to healthcare services combined could exacerbate the cancer disparity in SA. The disparities noted were also observed in a study conducted in the United States, where the proportions of CRC incidence were 59% for Whites and 30% for Blacks. However, the Annual Percentage Change (APC) for Blacks was 1.6 times higher than Whites [53]. The cause of variation of patterns and rates across population groups and sex may be attributed to socioeconomic disparities and differential risk behaviours [54, 55].

Stable ASIR and ASMR were observed among the Asian population group and females of the Mixed race population group over the study period. Stable ASIR but increasing ASMR was observed among Mixed race males. The Asian and Mixed race population groups account for 11% of the total population; thus, the numbers are likely too low to show a significant change in relation to other population groups [50, 56, 57]. However, the level of access to healthcare services among individuals of the Asian and Mixed race population groups remained fairly similar 4 and 10 years post-democracy (1994), a possible explanation for the rate of CRC diagnosis remaining stable [57, 58].

Among the White population group, the ASIR decreased from 2002 to 2007 for males and remained stable for females from 2002 to 2009, and then later increased from 2007 to 2014 for males, and from 2000 to 2014 for females. The ASMR remained stable throughout the study period for both males and females. The stabilisation or reduction observed between 2002 to 2009 may be partly attributed to the effects of withholding cancer reporting to NCR by private sector laboratories between 2005 and 2010 before the promulgation of Regulation 380 of the National Health Act no 61 of 2003 [59, 60]. An estimated 16% of the SA population seeks care in the private health sector, of which 92% are Whites, and hence the ASIR trends mimic the reporting levels during and few years after the data withholding period [60]. The stable ASMR was expected among the White population group, as 88% of the White population group have access to adequate healthcare services provided by the private healthcare sector [52]. The screening rates are higher than other population groups, which translates into early detection and treatment, and ultimately better prognosis and lower death rates, hence the stable mortality pattern observed [61, 62].

A consistent increase in ASIR and ASMR was observed among the Black population group throughout the study period. This may be explained by the rapid transition of the Black population group’s socioeconomic status and change in lifestyle [63, 64]. The increased adoption of western lifestyles and consequently, dietary changes as well as other risk factors for CRC may have led to an increase in CRC incidence among Black South Africans. This phenomenon is also observed by other African countries experiencing rapid westernisation [64, 65]. Secondly, the incidence pattern may be attributed to an increase in cancer reporting for the Black population group, which constitutes > 80% of the total SA population that was excluded from accessing quality healthcare services pre-democracy [49, 57, 66]. The ASMR trends observed may be explained by inequities in socioeconomic status differences. There is a general lack of access to quality healthcare services, late diagnosis of cancer, and limited treatment options available for poorer South Africans, as only 8% the Black population group can afford private medical insurance in SA [52, 67].

### Limitations

This study had several limitations. The incidence data used in this study was derived from the NCR. The NCR is a passive pathology-based cancer registry that collects histological, haematologically, and cytologically diagnosed cancer cases; therefore, CRC cases not diagnosed through laboratory means are excluded [18]. Between 2005 and 2007, cancer case reporting and data collection from private health facilities were limited due to concerns around confidentiality and the lack of legislature support on cancer reporting/surveillance in SA [60]. This may cause an underreporting of CRC incidence disproportionately amongst certain population groups that predominately utilised private healthcare facilities such as the non-Black population groups [60]. The lack of cancer staging in both the NCR and STATS SA data makes it more challenging to explore disparities in cancer incidence and mortality across population groups.

The data for cancer mortality was derived from STATS SA. Underreporting of death was likely across all population groups. In 2007, the completeness of mid-year estimates and vital statistics was below 90% as found by an evaluation [68]. This underreporting occurs because of delayed reporting, ill-defined causes of death, and misreporting or misclassification of cause of death [69].

Despite these limitations, this is the first national study to report CRC ASIR and ASMR trends in SA. Improvements in the accuracy of reporting will occur with more triangulation of data sources. Secondly, the data sources used are the primary source of cancer statistics in SA. This study has reported the CRC ASIR and ASMR, which were comparable with other rates reported by previous regional and international studies [70, 71].

## Conclusion

The increase in CRC ASIR and ASMR is an indication of the epidemiological transition of disease burden in SA with increasing HDI. The disparities across population groups, in particular, the consistent and rapid increase in ASIR among the Black population group, requires further in-depth studies to delineate the factors driving the increasing rates. Programs for promoting healthier behaviours such as reducing alcohol intake, reducing tobacco smoking, and increasing physical activities are recommended to reduce the risk of CRC in SA. The inequalities can be bridged through universal health coverage, targeted screening, early detection, and high-quality cancer care provision, especially for previously disadvantaged population groups. Enhancement of cancer surveillance, in particular, population-based cancer registration is required to produce better quality cancer data for in-depth exploratory research to better inform policies and interventions for cancer prevention and control in SA.

## Data Availability

The datasets used and/or analysed during the current study are publicly available from source and/or from the corresponding author on reasonable request. The data for colorectal incidence can be found on the South African National Cancer Registry’s website (www.ncr.ac.za). The colorectal mortality data provided by STATS SA can be found on their interactive website (http://nesstar.statssa.gov.za:8282/webview/).

## Abbreviations

AAPC: Average annual percentage change
APC: Annual percentage change
ASIR: Age-standardised incidence rate
ASMR: Age-standardised mortality rate
CRC: Colorectal Cancer
HDI: Human development index
NCR: South African National Cancer Registry
SA: South Africa
STATS SA: Statistics South Africa

## References

[1] Bray F, Ferlay J, Soerjomataram I, Siegel RL, Torre LA, Jemal A (2018). Global cancer statistics 2018: GLOBOCAN estimates of incidence and mortality worldwide for 36 cancers in 185 countries. CA Cancer J Clin.

[2] International Agency for Research on Cancer World Health Organization. GLOBOCAN Cancer Fact Sheets: lung Cancers. 2012. Available from: http://globocan.iarc.fr/Pages/fact_sheets_cancer.aspx. [cited 2018 Jun 15]

[3] Wild CP, BWS EW (2020). World Cancer Report: Cancer Research for Cancer Prevention.

[4] WHO (2019). GLOBOCAN 2018 REPORT WHO Africa (AFRO).

[5] World Health organisation. The Global Cancer Observatory -South Africa. WHO, Press vol. 097. Lyon, France. 2019. http://globocan.iarc.fr/Default.aspx.

[6] National Cancer Registry. National Cancer Registry 2016 South Africa. National Cancer Regsirty Statistics 2016. 2016. Available from: https://www.nicd.ac.za/centres/national-cancer-registry/ [cited 2020 Aug 1].

[7] Brand M, Gaylard P, Ramos J (2018). Colorectal cancer in South Africa: an assessment of disease presentation, treatment pathways and 5-year survival. S Afr Med J.

[8] Mccabe M, Perner Y, Magobo R, Mirza S, Penny C (2019). Descriptive epidemiological study of South African colorectal cancer patients at a Johannesburg hospital academic institution.

[9] Allemani C, Matsuda T, Di Carlo V, Harewood R, Matz M, Nikšić M, et al. Global surveillance of trends in cancer survival 2000–14 (CONCORD-3): analysis of individual records for 37 513 025 patients diagnosed with one of 18 cancers from 322 population-based registries in 71 countries. Lancet. 2018;391(10125):1023–1075. Available from: http://www.thelancet.com/article/S0140673617333263/fulltext [cited 2021 Jan 18] 10.1016/S0140-6736(17)33326-3 PMC5879496 29395269

[10] UN. Human Development Indices and Indicators. 2018 Statistical Update. United Nations Dev Program. 2018;27(4):123. Available from: http://www.hdr.undp.org/sites/default/files/2018_human_development_statistical_update.pdf%0Ahttp://hdr.undp.org/en/2018-update

[11] Crosbie AB, Roche LM, Johnson LM, Pawlish KS, Paddock LE, Stroup AM (2018). Trends in colorectal cancer incidence among younger adults—disparities by age, sex, race, ethnicity, and subsite. Cancer Med.

[12] American Cancer Society. Colorectal Cancer Facts&Figures 2017–2019. Am Cancer Soc. 2017:1–40 Available from: http://www.ncbi.nlm.nih.gov/pubmed/24225001.

[13] Davison P. The World Bank South Africa Overview. 2010. Available from: https://www.worldbank.org/en/country/southafrica/overview [cited 2020 Aug 13]

[14] Assessment A. Overcoming poverty and inequality in South Africa an Assessment of drivers, Constraints and Opportunities.

[15] Ataguba JE, Akazili J, McIntyre D. Socioeconomic-related health inequality in South Africa: Evidence from General Household Surveys. Int J Equity Health. 2011;10:48. Available from: /pmc/articles/PMC3229518/?report=abstract. [cited 2020 Aug 13] 10.1186/1475-9276-10-48 PMC3229518 22074349

[16] Coovadia H, Jewkes R, Barron P, Sanders D, McIntyre D (2009). The health and health system of South Africa: historical roots of current public health challenges. Lancet.

[17] Gilson L, McIntyre D (2007). Post-apartheid challenges: household access and use of health care in South Africa. Int J Health Serv.

[18] Singh E, Ruff P, Babb C, Sengayi M, Beery M, Khoali L, et al. Establishment of a cancer surveillance programme: the South African experience, Lancet Oncol; 2015. Vol. 16. p. e414–e421. Available from: http://www.ncbi.nlm.nih.gov/pubmed/26248849. NIH Public access. [cited 2017 Dec 4] 10.1016/S1470-2045(15)00162-X PMC4591917 26248849

[19] Fritz April, Percy Constance, Jack Andrew, Shanmugaratnam Kanagaratnam, Sobin LH et al. International classification of diseases for oncology / editors, April Fritz ... [et al.], 3rd ed. World Heal Organ. 2000; Available from: https://apps.who.int/iris/handle/10665/42344

[20] Boniol M, Heanue M. Chapter 7: age-standardisation and denominators. Cancer Incid Five Cont Vol IX. 2007:99–101.

[21] Segi M, Fujisaku S, Kurihara M. Geographical observation on cancer mortality by selected sites on the basis of standardised death rate. Gan. 1957;48(2):219–225. Available from: http://www.ncbi.nlm.nih.gov/pubmed/13474150 [cited 2019 Jul 8]. 13474150

[22] Vaughan AS, Kramer MR, Waller LA, Schieb LJ, Greer S, Casper M. Comparing methods of measuring geographic patterns in temporal trends: An application to county-level heart disease mortality in the United States, 1973 to 2010. Ann Epidemiol. 2015;25(5):329–335.e3. Available from: /pmc/articles/PMC4397179/?report=abstract. [cited 2021 Jan 17] 10.1016/j.annepidem.2015.02.007 PMC4397179 25776848

[23] Bray F, Guilloux A, Sankila R, Parkin DM (2002). Practical implications of imposing a new world standard population. Cancer Causes Control.

[24] Armitage P, Doll R (1954). The age distribution of cancer and a multi-stage theory of carcinogenesis. Br J Cancer.

[25] Kim HJ, Fay MP, Feuer EJ, Midthune DN. Permutation tests for joinpoint regression with applications to cancer rates. Stat Med. 2000;19(3):335–351. Available from: http://www.ncbi.nlm.nih.gov/pubmed/10649300. [cited 2019 Jul 9] 10.1002/(sici)1097-0258(20000215)19:3<335::aid-sim336>3.0.co;2-z 10649300

[26] National Cancer Institute (NCI). Joinpoint Help Manual. 2016:1–147. Available from: https://seer.cancer.gov/seerstat/ [cited 2021 Jan 17]

[27] American Cancer Society. Colorectal Cancer Facts & Figures 2020–2022. Am Cancer Soc Inc. 2020:1–32.

[28] Kim SE, Paik HY, Yoon H, Lee JE, Kim N, Sung MK. Sex- and gender-specific disparities in colorectal cancer risk. World J Gastroenterol. 2015;21(17):5167–5175. Available from: http://www.wjgnet.com/1007-9327/full/v21/i17/5167.htm [cited 2020 Feb 7] 10.3748/wjg.v21.i17.5167 PMC4419057 25954090

[29] White A, Ironmonger L, Steele RJC, Ormiston-Smith N, Crawford C, Seims A. A review of sex-related differences in colorectal cancer incidence, screening uptake, routes to diagnosis, cancer stage and survival in the UK. BMC Cancer. 2018;18(1):906. Available from: https://bmccancer.biomedcentral.com/articles/10.1186/s12885-018-4786-7 [cited 2020 Feb 7] 10.1186/s12885-018-4786-7 PMC6149054 30236083

[30] Laiyemo AO, Badurdeen D, Kibreab A, Scott VF, Lee EL, Brim H, et al. Su1677 Sex Disparity in Colorectal Cancer Burden Among Us Adults: The Role of Colon Cancer Screening Compliance. Gastrointest Endosc. 2017;85(5):AB390–AB391. Available from: https://linkinghub.elsevier.com/retrieve/pii/S0016510717310957 [cited 2020 Feb 7]

[31] NCR. National Cancer Registry 2012 annual report. 2012. Available from: http://www.nioh.ac.za/wp-content/uploads/2018/03/NCR-2012-results.pdf [cited 2019 Sep 3]

[32] NCR. National Cancer Registry 2013 annual report. 2013; Available from: http://www.nioh.ac.za/wp-content/uploads/2018/03/2013NCR.pdf [cited 2019 Sep 3]

[33] NCR (2014). National Cancer Registry 2014 annual report.

[34] Katsidzira L, Gangaidzo I, Thomson S, Rusakaniko S, Matenga J, Ramesar R. The shifting epidemiology of colorectal cancer in sub-Saharan Africa. Lancet Gastroenterol Hepatol. 2017;2(5):377–383. Available from: http://www.ncbi.nlm.nih.gov/pubmed/28397702 [cited 2019 Oct 1]. 10.1016/S2468-1253(16)30183-2 28397702

[35] Wei EK, Colditz GA, Giovannucci EL, Wu K, Glynn RJ, Fuchs CS (2017). A comprehensive model of Colorectal Cancer by risk factor status and subsite using data from the nurses’ Health study. Am J Epidemiol.

[36] Wolf AMD, Fontham ETH, Church TR, Flowers CR, Guerra CE, LaMonte SJ, et al. Colorectal cancer screening for average-risk adults: 2018 guideline update from the American Cancer Society. CA Cancer J Clin. 2018;68(4):250–281. Available from: http://doi.wiley.com/10.3322/caac.21457 [cited 2019 Jul 31] 10.3322/caac.21457 29846947

[37] Katsidzira L, Thomson SR, South African Gastroenterology Society. South African Gastroenterology Review. In House Publications; 2015. Vol. 13. 25–28 p. Cape Town, South Africa. Available from: https://journals.co.za/content/medgas/13/2/EJC174015 [cited 2019 Oct 1].

[38] Vajdic CM, MacInnis RJ, Canfell K, Hull P, Arriaga ME, Hirani V (2018). The future Colorectal Cancer burden attributable to modifiable behaviors: a pooled cohort study. JNCI Cancer Spectr.

[39] OECD. “Survival and mortality for colorectal cancer”, in Health at a Glance 2015. Paris: OECD Indicators, OECD Publishing; 2015. 10.1787/health_glance-2015-55-en.

[40] Magaji BA, Moy FM, Roslani AC, Law CW. Survival rates and predictors of survival among colorectal cancer patients in a Malaysian tertiary hospital. BMC Cancer. 2017;17(1):339. Available from: http://bmccancer.biomedcentral.com/articles/10.1186/s12885-017-3336-z [cited 2019 Oct 1] 10.1186/s12885-017-3336-z PMC5437641 28521746

[41] Aryaie M, Roshandel G, Semnani S, Asadi-Lari M, Aarabi M, Vakili MA, et al. Predictors of Colorectal Cancer Survival in Golestan, Iran: A Population-based Study. Epidemiol Health. 2013;35:e2013004. Available from: http://e-epih.org/journal/view.php?doi=10.4178/epih/e2013004 [cited 2019 Oct 1] 10.4178/epih/e2013004 PMC3691365 23807907

[42] Carethers JM. Clinical and Genetic Factors to Inform Reducing Colorectal Cancer Disparitites in African Americans. Front Oncol. 2018;8:531. Available from: http://www.ncbi.nlm.nih.gov/pubmed/30524961. [cited 2019 Oct 1] 10.3389/fonc.2018.00531 PMC6256119 30524961

[43] Watlington AT, Byers T, Mouchawar J, Sauaia A, Ellis J. Does having insurance affect differences in clinical presentation between Hispanic and non-Hispanic white women with breast cancer? Cancer. 2007;109(10):2093–2099. Available from: http://doi.wiley.com/10.1002/cncr.22640 [cited 2019 Oct 1] 10.1002/cncr.22640 17420982

[44] Quintana JM, Antón-Ladislao A, González N, Lázaro S, Baré M, Fernández-de-Larrea N (2018). Predictors of one and two years’ mortality in patients with colon cancer: A prospective cohort study. PLoS One.

[45] Gimeno Garcia AZ, Hernandez Alvarez Buylla N, Nicolas-Perez D, Quintero E (2014). Public awareness of Colorectal Cancer screening: knowledge, attitudes, and interventions for increasing screening uptake. ISRN Oncol.

[46] Gede N, Reményi Kiss D, Kiss I. Colorectal cancer and screening awareness and sources of information in the Hungarian population. BMC Fam Pract. 2018;19(1):106. Available from: https://bmcfampract.biomedcentral.com/articles/10.1186/s12875-018-0799-1 [cited 2019 Oct 1] 10.1186/s12875-018-0799-1 PMC6026511 29960585

[47] Stupart DA, Goldberg PA, Algar U, Ramesar R. Surveillance colonoscopy improves survival in a cohort of subjects with a single mismatch repair gene mutation. Color Dis. 2009;11(2):126–130. Available from: http://www.ncbi.nlm.nih.gov/pubmed/19143775. [cited 2019 Oct 1] 10.1111/j.1463-1318.2008.01702.x 19143775

[48] Anderson DW, Goldberg PA, Algar U, Felix R, Ramesar RS. Mobile colonoscopic surveillance provides quality care for hereditary nonpolyposis colorectal carcinoma families in South Africa. Color Dis. 2007;9(6):509–514. Available from: http://www.ncbi.nlm.nih.gov/pubmed/17477847. [cited 2019 Oct 1] 10.1111/j.1463-1318.2006.01172.x 17477847

[49] Release S (2019). Mid-year population estimates.

[50] South African Human Rights Commission. Equality Report : Achieving substantive economic equality through rights-based radical socio-economic transformation in South Africa 2017/18. 2018; Available from: https://www.sahrc.org.za/home/21/files/SAHRC Equality Report 2017_18.pdf.

[51] Mcintyre D. Private sector involvement in funding and providing health services in South Africa : Implications for equity and access to health care. Equinet Discuss Pap 84. 2010:1–52.

[52] Statistics South Africa. Use of health facilities and levels of selected health conditions in South Africa: Findings from the General Household Survey, 2011. Vol. 05, Report No. 03–00–05 (2011). 2011. 22 p. Available from: http://www.statssa.gov.za/publications/report-03-00-05/report-03-00-052011.pdf

[53] Van Beck KC, Jasek J, Roods K, Brown JJ, Farley SM, List JM (2018). Colorectal Cancer incidence and mortality rates among New York City adults ages 20–54 years during 1976–2015. JNCI Cancer Spectr.

[54] Lee W, Nelson R, Mailey B, Duldulao MP, Garcia-Aguilar J, Kim J. Socioeconomic Factors Impact Colon Cancer Outcomes in Diverse Patient Populations. J Gastrointest Surg. 2012;16(4):692–704. Available from: http://www.ncbi.nlm.nih.gov/pubmed/22258868. [cited 2019 Jul 31] 10.1007/s11605-011-1809-y 22258868

[55] Tawk R, Abner A, Ashford A, Brown CP. Differences in colorectal cancer outcomes by race and insurance. Int J Environ Res Public Health. 2015;13(1):ijerph13010048. Available from: http://www.ncbi.nlm.nih.gov/pubmed/26703651. [cited 2019 Jul 31] 10.3390/ijerph13010048 PMC4730439 26703651

[56] MartinVA. General Household survey 2017 Statistical Release. J Thorac Cardiovasc Surg. 2015;149(1):A33–A34. Available from: www.statssa.gov.za [cited 2020 Aug 13]

[57] Delobelle P. The health system in South Africa. Historical perspectives and current challenges. S Afr Focus Econ Polit Soc Iss. 2013:159–205.

[58] R Lalloo, M J Smith, N G Myburgh GCS. Access to health care in South Africa — the influence of race and class. S Afr Med J. 2012;102(7):599–601. Available from: http://www.samj.org.za/index.php/samj/article/view/5789/4270 [cited 2019 Dec 2] 15352587

[59] Gazette G. GOVERNMENT NOTICE. Heal (San Fr. 2008;(10505):2–6.

[60] Singh E, Underwood JM, Nattey C, Babb C, Sengayi M, Kellett P. South African National cancer registry: Effect of withheld data from private health systems on cancer incidence estimates. South African Med J. 2015;105(2):107–109. Available from: https://www.ncbi.nlm.nih.gov/pmc/articles/PMC4591919/pdf/nihms724320.pdf [cited 2017 Dec 4] 10.7196/SAMJ.8858 PMC4591919 26242527

[61] Brandt HM, Dolinger HR, Sharpe PA, Hardin JW, Berger FG. Relationship of colorectal cancer awareness and knowledge with colorectal cancer screening. Color cancer. 2012;1(5):383–396. Available from: http://www.ncbi.nlm.nih.gov/pubmed/26257828. [cited 2019 Oct 2] 10.2217/crc.12.45 PMC4529290 26257828

[62] Singh GK, Jemal A. Socioeconomic and Racial/Ethnic Disparities in Cancer Mortality, Incidence, and Survival in the United States, 1950-2014: Over Six Decades of Changing Patterns and Widening Inequalities. J Environ Public Health. 2017;2017:2819372. Available from: http://www.ncbi.nlm.nih.gov/pubmed/28408935. [cited 2020 Feb 10] 10.1155/2017/2819372 PMC5376950 28408935

[63] Irabor D. Emergence of colorectal cancer in West Africa: Accepting the inevitable. Niger Med J. 2017;58(3):87. Available from: http://www.nigeriamedj.com/text.asp?2017/58/3/87/234076 [cited 2019 Oct 1] 10.4103/0300-1652.234076 PMC6009139 29962648

[64] Wallace M, Pentz-kluyts M. Addressing obesity as a risk factor for Cancer in South Africa Size does matter. Cancer Control. 2017;(7):93–98. Available from: https://www.cansa.org.za/addressing-obesity-as-a-risk-factor-for-cancer-in-south-africa-size-does-matter/ [cited 2019 Oct 1]

[65] Irabor DO. Emergence of Colorectal Cancer in West Africa: Accepting the Inevitable. Niger Med J. 2017;58(3):87–91. Available from: http://www.ncbi.nlm.nih.gov/pubmed/29962648. [cited 2019 Oct 1] 10.4103/0300-1652.234076 PMC6009139 29962648

[66] Presidency of the Government of South Africa. Twenty Year Review (1994-2014). 2014;166.

[67] Ataguba JE, McIntyre D (2012). Paying for and receiving benefits from health services in South Africa: is the health system equitable?. Health Policy Plan.

[68] Joubert J, Rao C, Bradshaw D, Vos T, Lopez AD (2013). Evaluating the quality of National Mortality Statistics from civil registration in South Africa, 1997-2007. PLoS One.

[69] Release S. Mortality and causes of death in South Africa, 2016: Findings from death notification. Statistical Release P0309.3. 2018:138. Available from: http://www.statssa.gov.za/publications/P03093/P030932016.pdf

[70] Arnold M, Sierra MS, Laversanne M, Soerjomataram I, Jemal A, Bray F (2017). Global patterns and trends in colorectal cancer incidence and mortality. Gut..

[71] Graham A, Adeloye D, Grant L, Theodoratou E, Campbell H. Estimating the incidence of colorectal cancer in Sub-Saharan Africa: A systematic analysis. J Glob Health. 2012;2(2):020404. Available from: http://www.ncbi.nlm.nih.gov/pubmed/23289079. [cited 2020 Feb 10] 10.7189/jogh.02.020204 PMC3529315 23289079

